# Retraction Note: Meta-analysis of the effect of percutaneous coronary intervention on chronic total coronary occlusions

**DOI:** 10.1186/s13019-015-0235-8

**Published:** 2015-03-27

**Authors:** Ruogu Li, Shuansuo Yang, Lei Tang, Yiqing Yang, Hui Chen, Shaofeng Guan, Wenzheng Han, Hua Liu, Jinjie Dai, Qian Gan, Weiyi Fang, Xinkai Qu

**Affiliations:** 1 Department of Cardiology, Shanghai Chest Hospital, Shanghai Jiaotong University, NO.241, Huaihai Xi Road, Xuhui District, Shanghai, 200030 China; 2 Department of Cardiology, Central Hospital, Fengxian District, Shanghai, 201400 China; 3 School of Medical Graduate, Shanghai Jiaotong University, Shanghai, 200025 China; 4 Department of Cardiovascular Research, Shanghai Chest Hospital, Shanghai Jiaotong University, Shanghai, 200030 China

## Retraction

The Publisher and Editor regretfully retract this article [1] because the peer-review process was inappropriately influenced and compromised. As a result, the scientific integrity of the article cannot be guaranteed. A systematic and detailed investigation suggests that a third party was involved in supplying fabricated details of potential peer reviewers for a large number of manuscripts submitted to different journals. In accordance with recommendations from COPE we have retracted all affected published articles, including this one. It was not possible to determine beyond doubt that the authors of this particular article were aware of any third party attempts to manipulate peer review of their manuscript.
